# Time to Act: Lessons Learnt from the First Pilot School-Based Intervention Study from Lebanon to Prevent and Reduce Childhood Obesity

**DOI:** 10.3389/fpubh.2015.00056

**Published:** 2015-04-15

**Authors:** Carla Habib-Mourad, Lilian A. Ghandour

**Affiliations:** ^1^Department of Nutrition and Food Sciences, Faculty of Agriculture and Food Sciences, American University of Beirut, Beirut, Lebanon; ^2^Department of Epidemiology and Population Health, American University of Beirut, Beirut, Lebanon

**Keywords:** obesity, intervention studies, school children, Lebanon, overweight

## Abstract

Today, childhood overweight and obesity are serious public health problems that the world faces. Obese children suffer from both short-term and long-term health consequences, and poorer adult health. Despite the rising prevalence of childhood obesity in the Eastern Mediterranean Region, including Lebanon, no intervention research studies have been undertaken. This paper summarizes the main challenges and lessons learned emanating from the first evidence-based multicomponent school intervention aimed at promoting Healthy Eating and Physical Activity in Lebanese School children (Health-E-PALS). Health-E-PALS, which includes three components (class curriculum, family involvement, and food service) and relies on interactive fun learning activities, achieved an increase in students’ nutritional knowledge and self-efficacy, and a decrease in their purchase and consumption of high energy dense snacks and beverages. Recommendations for future school-based programs are also highlighted.

## Childhood Obesity and the Eastern Mediterranean Region

Overweight and obesity are serious public health problems posing one of the most difficult challenges for the twenty-first century. Globally, the prevalence of childhood obesity (2–19 years) has been on the rise at a disturbing pace with current rates almost 10 times higher than those in the 1970s (1). The rise has been occurring at a faster pace in economically developed countries and in urbanized populations (1), including countries within the Middle East and the Arabian Peninsula regions. According to a recent review by Musaiger (2), the number of overweight and obese school children has reached alarming levels in most Eastern Mediterranean Region (EMR) countries, reaching an unprecedented number of 41.7 million in 2010. Childhood overweight and obesity rates in the EMR have been estimated to be second to the US, exceeding that of European countries (3). This high prevalence of overweight and obesity among school children in the EMR has been illustrated in several school-based studies, particularly in countries with high-income (4). Overweight estimates (BMI ≥85th percentile) (1) in various countries vary as follows among school children aged 2–18 years: 11–28.8% in the Kingdom of Saudi Arabia (KSA) (5–7), 12.3–34.5% in United Arab Emirates (UAE) (8, 9), 20.2–31.8% in Kuwait (10–12), and 15.7–29.4% in Bahrain (13, 14). The prevalence of obesity (BMI ≥95th percentile) (1) among school children varies between 5 and 26.4% in KSA (5–7, 13), 3 and 21.6% in UAE (8, 9), 13.1 and 16.8% in Kuwait (10–12), and 12.7 and 19.4% in Bahrain (13, 14).

Despite the methodological differences, some general observations/conclusions can be made for the EMR region including: (1) overweight is still more common than obesity in both genders (9, 10); (2) gender differences are inconclusive, whereby in some countries, rates of overweight and obesity are higher in males (8, 15), while in others the opposite is true (10); and (3) overweight and obesity may be on the rise in some countries such as Lebanon (a middle–high income country), whereby a rapid increase in BMI across all sex and age groups was observed between 1997 and 2009, and a significant increase in obesity among 6- to 19-year olds (7.3% in 1997 versus 10.9% in 2009) (16). In a school-based survey in 2007, 6.6% of the public school students (aged 11–18 years) were identified as obese and 20.5% were at risk of obesity (17); estimates were 7.5% and 24.4% in private school students, respectively (18).

Childhood obesity is one of the strongest predictors of obesity in adulthood (19). It has also been strongly and consistently associated with several ill-health conditions and diseases, in addition to important psychosocial consequences such as decreased self-confidence and increased depression as a result of the potential and uncalled for stigmatization of obese children and adolescents (20). The global concern over the development of metabolic syndrome (MS) abnormalities (i.e., elevated waist circumference, high triglyceride, and low high-density lipoprotein cholesterol levels) in children is also pertinent to EMR countries. Prevalence of the MS was estimated at 13% in overweight and obese Emirati boys (21), 26.4% among obese children in Lebanon, and as high as 41.9% in obese children and adolescents in Iran (22).

## Public Health Interventions Targeting Childhood Obesity in EMR: A Case-Study from Lebanon

In light of the epidemiological evidence, multicomponent interventions, policies, and nutritional strategies to promote weight control and physical activity in the EMR become integral to curbing the prevalence of childhood obesity and preventing its ill-health consequences. To our knowledge, and based on a thorough online search, no school-based intervention studies on promoting healthy eating or encouraging physical activity have been published from the region.

In response to the absence of such interventions, coupled with the obesity epidemic in Lebanon, the Healthy Eating and Physical Activity in Lebanese School children (Health-E-PALS) project was launched in 2009. It is worth noting that the integrated health curriculum in Lebanon incorporating nutrition education to students in grades 4 and 5 as part of “The life sciences and biology” component of education comprises only of one chapter covering basic information about food groups and classes of nutrients. Health-E-PALS is a theory and evidence-based multicomponent culturally appropriate pilot school-based intervention, which addresses nutrition awareness and physical activity among children aged 9–11 years.

The school-based intervention was developed and piloted by the lead author (Carla Habib-Mourad), who personally delivered the sessions with the help of a research assistant, and in the presence of the teacher. Health-E-PALS targets variety of energy balance health behaviors and employs carefully planned, theory-based multidisciplinary approaches (23) as an effective strategy to preventing and reducing childhood obesity (24, 25). Detailed description of the development of Health-E-PALS is provided elsewhere (26). Briefly, Health-E-PALS incorporated several of the success factors reported in effective interventions to prevent obesity in school children (27). Specifically, it (1) used fun and interactive class activities to promote healthy eating and physical activity as part of the school curriculum; (2) emphasized on afterschool active play as part of increasing the frequency of physical activity; (3) enhanced the variety of good nutritional quality foods within the school food service; and (4) solicited support from the home environment to encourage the adoption of healthier food and activity habits. A total of 12 classroom sessions were delivered, 45 min each over a 12-week period (approximately 3 months). Each session consisted of a 10- to 15-min interactive informative discussion, followed by a 30-min activity: game and/or food preparation.

Health-E-PALS was effective in reducing purchase and consumption of high energy dense snacks and beverages and increasing students’ nutritional knowledge and self-efficacy (27). Like any intervention study, the challenges were numerous; some were perceived by the research team during implementation, while others were shared by a sub-sample of the students, their parents, and teachers during 13 follow-up focus group discussions (FGDs) and 2 in-depth interviews involving a total of 82 participants.

In light of the importance of implementing additional future interventions, this paper aims at providing a synthesis of the main challenges and lessons learnt, some of which may be unique to the Lebanese context but many of which may be applicable and important to other similar contexts.

### Getting the schools on board

Perhaps one of the biggest challenges was getting the school principals to partake in the study and implement the program throughout the academic year. This was not so much of an issue with public schools since we had approached and received the approval of the Lebanese Ministry of Education and Higher Education (MEHE), which in Lebanon automatically secures public schools’ participation. This of course may be true in one context and not the other, so the lesson learnt is to secure the approval and endorsement of all relevant authorities, whether political or educational, prior to approaching the schools to streamline the process. Recruitment of private schools was more challenging since each had to be approached and persuaded separately. Irrespective, endorsement of a governmental body and support via various media outlets (i.e., television shows, health blogs, and magazines) may prove useful in encouraging all schools’ participation.

Another point to consider relates to the readiness of schools to participate as controls in the research study. When informed that they may be randomized into a control group, some schools were reluctant to partake in the pilot intervention study. When faced with this challenge, we resolved it by assuring the school principals that they would be provided with an extensive training and all educational material at the end of the intervention (if they were selected into the control group).

### Ensuring a fun, interactive, integrated, and culturally relevant educational component

One factor that helped us persuade school principals to partake in the program is the fact that our educational component was integrative and interdisciplinary; in other words, the content of the intervention material was developed in such a way to make it possible to integrate into most classes (26). Nutrition sessions were integrated into various classroom subjects during a regular school day. For example, students used the food portions session to practice fractions during math class, and breakfast planning took place during writing exercises in English or Arabic classroom sessions. While the majority of administrators welcomed the integrative approach and felt it would avoid any excess burden on the existing school curriculum, some school principals, however, were reluctant to integrate the program into math, language, or science classes, and preferred to give-up an art or physical education class instead. However, during our process evaluation and observations, we learnt that integrating the program sessions into the curriculum also gave better results; meaning, students seemed more engaged and knowledgeable. Integrating the sessions into the existing school curriculum also helps sustain the program over a longer period and across several grade levels. Indeed, the importance and need for follow-up in young children was raised by all interviewed.

The fun aspect of the educational activities helped improve student participation, and subsequently outcome measures. From our discussions with students, we learnt that their active involvement during in-class food preparation activities was a key factor to enhancing their skills and self-confidence in choosing and preparing healthy foods. This sentiment was also shared by the parents who felt that their children’s interest in the intervention was largely due to the fact that the program was not a lesson to memorize or to be tested on. The games and activities encouraged their children to learn and change their habits. Reinforcements such as praise and tokens (e.g., balls, jumping ropes, pedometers…) were also perceived as helpful. Parents also acknowledged the positive messages about food choices, which were quite moderate, with no labeling of food products as “good” or “bad.” This was the intention of the program, which focused on the promotion of healthy food choices and an active lifestyle rather than the achievement of an ideal body weight. This approach is conducive to lessening the chance of stigmatization of overweight children and of contributing to eating disorders (28).

Unfortunately, sometimes “fun activities” were hindered by structural barriers; for instance, increased physical activity was often impeded by having very small playgrounds at schools, and/or no play area near the students’ homes. When possible, however, students felt that a higher frequency of physical activity sessions during school hours would benefit the program and its intended outcomes (most school curricula offer physical education twice per week).

Quite important is ensuring that all materials used throughout the program are developed and tailored to the relevant context; in our case, all materials were in line with the Lebanese culture and traditions, and featured local traditional foods in most games, visual aids, and recipes. Similar interventions in other countries/contexts must therefore ensure relevance and adapt the food items/games along with other educational material to meet the needs and interests of the children in that particular country/context.

### Working closely with the school shop owners

Typically, foods and drinks available to students in the sampled Lebanese school shops included chips, candy bars, sweetened drinks as well as ready prepared sandwiches, traditional Lebanese pastries, croissants, and donuts. Unfortunately, fresh juices, fruits, and vegetables were not made available to the students. Involving school shop owners in the program by recommending that they serve a healthier list of snacks and drinks was quite a challenge in some schools, especially when the shop was operated by an external party. Shop owners were worried about their revenues, and we had to reassure them that selling healthy foods on the long run will not jeopardize their income. Besides recommending healthier choices, we also worked with the vendors on ways to work with limited space, and suggest a list of food products with long shelf life (i.e., do not need refrigeration, simple, and safe packaging). Providing healthier choices was particularly challenging in public schools, as private schools were in a better disposition financially to improve their service level. Economically disadvantaged schools may therefore benefit greatly from governmental subsidies to help provide healthier choices.

### Engaging parents all the way

Involving the parents at every stage is pivotal for the program’s success and for ensuring that the children are being provided with a home environment supportive of healthy lifestyle behaviors. The program, Health-E-PALS, for instance, encourages students to enhance the quality of their lunch box so as to include at least one fruit or vegetable portion and not more than one high energy dense snack. Quite often, however, and especially at the primary school level, it is the parents who prepare the lunch box, which typically consists of sandwiches and convenience foods. Parental meetings were held for that purpose (where a healthy breakfast was served), but securing parental attendance was another challenge, which we overcame by inviting the parents to attend their children’s performances (health fairs, breakfast prepared by students…). We also continuously engaged the parents by sharing the educational sessions and sending special home-packs after each session (consisting of a summary of the major points covered in class with a sample of the foods prepared at school). The take-home material was one way to help ensure the transfer of knowledge amidst non-compliance/poor attendance of parents in school-held meetings. It is worth noting, however, that parental attendance varied between families of different social, financial, and educational backgrounds. In public schools, it was hard to engage parents who had difficulty in arranging for transportation to/from school, or had younger children at home to take care of; in private schools, the reason for not attending the meetings was mostly because both parents were employed. Several meetings may therefore have to be planned to accommodate parents’ schedule.

Occasionally, program implementers may be faced with parental reluctance to enroll their child in a nutritional intervention; as one parent put it: “my child is fit, no need for a diet.” Of course, once the program is integrated into the school curriculum, this will no longer be an issue.

### Plan for longer project duration and sustainability

A unanimous suggestion among parents, teachers, and students was to increase the duration of the intervention, and to extend it preferably over the entire academic year. Indeed, the length of the intervention for the pilot project (3 months) was relatively short to expect major behavioral changes; for instance, despite the many positive changes, no differences were observed for frequency of physical activity or screen time habits or BMI. The integrative aspect makes it possible to extend it over a period of 9 months rather 3–6 months, particularly when the program is adopted and delivered by trained school staff. This would also resolve and overcome a main challenge that was faced, which was convincing some school administrators to implement a 3- to 4-month intervention. During the FGDs, teachers expressed interest and willingness to attend further training that would enable them to deliver the program independently. Teachers’ presence in the class when the lessons were being delivered was also found very useful especially in maintaining discipline. Still, teachers in public schools (where lower wages are earned) may be less enthusiastic to overburden themselves and their curriculum with additional educational material.

### Be prepared for unexpected events

During implementation, certain events may cause unanticipated delays, and the team must have a contingency plan. Lebanon, as is the case with several countries in the EMR, has experienced irregular bouts of political instability and insecurity, which in our case affected the timeline of implementation. Nonetheless, extra sessions were scheduled to make-up for the sessions that were canceled due to strikes and other security issues. This extended time frame for the intervention posed a problem to some schools as it coincided with students’ final exams. Integrating the intervention into the school curriculum may also override such unexpected delays.

## Conclusion

The prevalence of overweight and obesity in most countries of the EMR region has been described as “alarming,” and yet no known school-based or other nutritional interventions have been conducted to tackle the rise of the childhood obesity epidemic in the region. It is this lack of national strategy that prompted us to develop Health-E-PALS, the first evidence-based multicomponent culturally appropriate school intervention aimed at increasing nutrition awareness and physical activity with school children. Several evidence-informed recommendations can be made Table 1. First, the educational material must be informative but also fun, interactive, and relevant to the population. Integrating the program into a school curriculum would allow for longer periods of learning, observation, and re-assessment, and may accomplish more positive behavioral changes. Ownership of the program by the various stakeholders is probably one of the most key lessons learnt, so engaging the relevant ministries, schools administrators, school-based vendors, parents, and of course students is essential for streamlining the implementation of any program and ensuring its success.

**Table 1:** **Recommendations for establishing and sustaining a school-based nutritional program**.

Create a program that is fun, educational, interactive, and relevant to the context and its population; make sure all the materials are pre-tested and adapted to the local setting
Train school staff to minimize cost (human resources) and ensure sustainability of the program
Work closely with the food service establishments and canteens in schools to secure availability of healthy foods
Engage the parents throughout the entire process to help create a supportive home environment for healthier nutritional choices and lifestyle
Engage the local community, mayors, municipalities, health care facilities, community gyms to provide indirect support for the prevention

## Conflict of Interest Statement

The authors declare that the research was conducted in the absence of any commercial or financial relationships that could be construed as a potential conflict of interest.
